# Community engagement and the human infrastructure of global health research

**DOI:** 10.1186/1472-6939-15-84

**Published:** 2014-12-13

**Authors:** Katherine F King, Pamela Kolopack, Maria W Merritt, James V Lavery

**Affiliations:** Center for Ethical, Social, and Cultural Risk, LiKaShing Knowledge Institute, St. Michael’s Hospital, Toronto, Canada; Berman Institute of Bioethics, Johns Hopkins University, Baltimore, MD USA; Department of International Health (Health Systems Program), Johns Hopkins Bloomberg School of Public Health, Baltimore, MD USA; Dalla Lana School of Public Health and Joint Centre for Bioethics, University of Toronto, Toronto, Canada

**Keywords:** Community engagement, Ethics, Respect, Legitimacy, Global health

## Abstract

**Background:**

Biomedical research is increasingly globalized with ever more research conducted in low and middle-income countries. This trend raises a host of ethical concerns and critiques. While community engagement (CE) has been proposed as an ethically important practice for global biomedical research, there is no agreement about what these practices contribute to the ethics of research, or when they are needed.

**Discussion:**

In this paper, we propose an ethical framework for CE. The framework is grounded in the insight that relationships between the researcher and the community extend beyond the normal bounds of the researcher-research participant encounter and are the foundation of meaningful engagement. These relationships create an essential “human infrastructure” – a web of relationships between researchers and the stakeholder community—i.e., the diverse stakeholders who have interests in the conduct and/or outcomes of the research. Through these relationships, researchers are able to address three core ethical responsibilities: (1) identifying and managing non-obvious risks and benefits; (2) expanding respect beyond the individual to the stakeholder community; and (3) building legitimacy for the research project.

**Summary:**

By recognizing the social and political context of biomedical research, CE offers a promising solution to many seemingly intractable challenges in global health research; however there are increasing concerns about what makes engagement meaningful. We have responded to those concerns by presenting an ethical framework for CE. This framework reflects our belief that the value of CE is realized through relationships between researchers and stakeholders, thereby advancing three distinct ethical goals. Clarity about the aims of researcher-stakeholder relationships helps to make engagement programs more meaningful, and contributes to greater clarity about when CE should be recommended or required.

## Background

Biomedical research is increasingly globalized with ever more research conducted in low- and middle-income countries (LMICs). But the dominant paradigm remains essentially unchanged; the majority of funding coming from funding agencies and foundations in high-income countries (HIC) to support research programs and projects run by researchers in HIC institutions who conduct their research activities in LMIC settings [1]. The “background conditions”, of global inequality and injustice frame this research and raise a host of ethical concerns [2]. These conditions are further amplified by cultural and linguistic differences, a historical legacy of distrust and exploitation within the research enterprise, and concerns about scientific colonialism [3]. While these background conditions create significant challenges, they are challenges that need to be addressed and managed in the design and conduct of global biomedical research, which remains an important and fundamentally ethical pursuit, and essential to inform solutions to some of the world’s most pressing health problems.

One recurring theme in reporting about international biomedical research is that there is inadequate opportunity for research participants and affected communities to voice their concerns, and for researchers to acknowledge them and respond constructively [1–7]. Too often, these concerns go unaddressed and cause breakdowns in crucial relationships that, in turn, contribute to various types of failure, disappointment, or resentment, outcomes that perpetuate the historical injustices described above [8].

We argue that to address these problems, researchers, sponsors and funders need to invest in the human infrastructure of global health research: that web of relationships between researchers and the stakeholder community— the unique collection of diverse stakeholders who have interests in the conduct and/or outcomes of a given research project. Community Engagement (CE) is a promising strategy for cultivating this infrastructure. CE refers to a set of practices that help researchers establish and maintain relationships with the stakeholders to a research program [9]. While CE has become increasingly common in global health research, there is concern that the engagement is often not meaningful, particularly when it is mediated solely through Community Advisory Boards (CABs) [10–14]. To understand what makes CE meaningful, we have developed a framework to clarify what CE contributes to the ethical quality of research. Clarity about these ethical goals is critical to both improving CE practices, and to understanding when these practices are important to the ethical conduct of research.

To that end, we propose an ethical framework for CE. The framework is grounded in the insight that relationships between researchers and the community of stakeholders in a given global health research project are the foundation of meaningful engagement. We argue that it is primarily through these relationships that researchers are able to address three core ethical responsibilities: [1] identifying and managing non-obvious risks; [2] extending respect beyond the individual to the stakeholder community; and [3] building legitimacy for the research project. Although each of these ethical goals has received attention individually in the ethics of research, we propose that collectively they represent a coherent and comprehensive framework that clarifies the unique contributions of CE to global health research, and may serve as a useful reference for the on-going debate about how to evaluate the quality and impact of CE.

## Discussion

### Ethical goals of community engagement

#### Identifying and managing non-obvious risks

In current research ethics review procedures, investigators and Institutional Review Boards (IRBs) must delineate and minimize risks to participants--including physical, psychological, social, economic, and legal risks. However, what constitutes a risk, and which risks are deemed to be acceptable, and by whom, may not be obvious to remote researchers and their IRBs, who may be unfamiliar with ethically significant features of the specific research context. Indeed, the nature and distribution of some risks may not be obvious to potential research participants themselves [15].

Non-obvious risks—by definition—are not conveniently accessible to researchers through casual observation. For example, awareness of the cultural significance of blood and biological tissues, and the various ways in which related research practices might offend a community’s deeply-held beliefs about them, [16] and insights about what research practices might avoid or minimize the offense, is gained through dialogue and on-going communications with the stakeholder community [14]. If researchers do not understand the significance or implications of the research for stakeholders, they may fail to recognize what interests are at stake, and so may fail to take the necessary steps to protect those interests, thereby exposing participants and host communities to unnecessary risk [17]. The nature of the relationship between researchers and stakeholders, i.e., the extent to which they are able to develop a comfortable rapport and confidently place trust in one another, influences the extent to which researchers are able to identify non-obvious risks and take steps to mitigate them. Failure to understand and evaluate research from the perspective of the stakeholder community, including prospective research participants, can also jeopardize the scientific quality of the research by compromising recruitment, retention, and adherence to the protocol, as well as the acceptability and ultimate adoption of any technology under development [4].

#### Extending respect to the stakeholder community

CE is regularly presented as an important mechanism for researchers to demonstrate respect for communities, but the precise contribution of CE to respect remains unclear [10, 11, 18–20]. We propose that CE respects affected communities by acknowledging what is valuable or important about research to them, *as people*, rather than prospective participants or facilitators of the research project, and then acting in ways that express that recognition [21, 22].

Researchers respect stakeholders by first listening to them to understand their perspectives about the research and how it may affect their interests, and then acting in ways that express that recognition. The simple act of listening is the foundation of respect and at the heart of what makes CE meaningful. By listening to affected communities, the research team acknowledges the importance of the various stakeholders, their interests, and their moral standing to hold researchers morally accountable for research-related actions, risks, and burdens. Given the historical legacy of colonialism and exploitation in many host countries, where the interests of the population were systematically disregarded, listening to, acknowledging, and being responsive to stakeholders acquires great significance.

These actions also represent important gestures of respect because they communicate an appreciation, on the part of researchers, that they have an obligation to discover the ways in which their research might affect the interests of a wide range of stakeholders. In this way, researchers extend the conventional notion of respect for the autonomy of individual research participants to a more comprehensive account of respect for the interests of the host community.

#### Building legitimacy for the research project

The third ethical goal of CE is to build legitimacy for the research project. Legitimacy is a political concern about the justification of authority over groups of people [23]. It emerges as a concern for biomedical research with the recognition that research activities usually affect the interests of parties beyond the direct research participants, yet despite this broad reach, there are currently no set mechanisms to gauge the relevance or strength of these interests, or to be responsive to them. A range of considerations contribute to the legitimacy of a research project in the eyes of its stakeholders: the perceived social value of the research; the nature and extent of risks imposed on the community; the perceived trustworthiness of the researchers, sponsoring institutions and funders; the transparency with which the research is conducted; and the mechanisms of accountability between the research team and the affected community. CE contributes to each of these considerations by building the infrastructure needed to connect researchers and stakeholders, and it is through these relationships that concerns of legitimacy can be addressed [10, 11, 18–20, 22].

Legitimacy is built through both formal and informal processes. Formally, research must be reviewed and approved by regulatory authorities such as Research Ethics Committees. Informally, a project’s legitimacy is built largely through deliberation and discussion with stakeholders, including collaborations, institutional partners, various levels of government, local residents, informal authorities and anyone whose interests stand to be affected by the proposed research.

Through informal deliberative processes, stakeholders and researchers engage in the give-and-take of reasons around the important issues surrounding the project and the ultimate course of action can be properly justified to the parties involved [23]. Not all stakeholders can or must be involved in decisions in order to make them legitimate, nor is it necessary that all stakeholders interact in a common forum [23]. What is important, rather, is that there be meaningful efforts to understand the interests at stake for various stakeholders, and processes in place to air disagreements and discuss the concerns and interests of the stakeholder community.

The goal of such discussions is not to resolve all disagreement and reach consensus around the research. Given the diversity of stakeholders involved, complete consensus is unlikely. Rather, CE embodies a democratic ideal in which legitimacy emerges from deliberative processes through which disagreement is acknowledged and addressed, lines of accountability are established between the stakeholder community and researchers, and stakeholders are empowered to ask directly for justification regarding the trial’s conduct and management.

Recent open-release trials of genetically-modified mosquitoes to control Dengue virus transmission highlight the importance of differentiating the legitimacy that is achieved through the endorsement of formal and informal authorities in a research community. The approval of trials by legitimate government authorities, with limited consultation or engagement with other stakeholders in the host community, was severely criticized by a wide range of stakeholders. The stakeholders argued that government approval alone was insufficient for the trial to go forward as it did not adequately address the risks and benefits of the trial for different stakeholders [24]. These objections had enormous impact on public opinion about the trial and the authority of the government to approve the research independent of meaningful engagement with the wider public [25].

### Community engagement creates human infrastructure for biomedical research

The three ethical goals, described above, represent important ethical considerations that are not currently reflected in most research ethics guidelines and regulations governing global health research. Most notably, they are distinct in their emphasis on the perspective of stakeholders, as opposed to the institutional perspective of the committees or regulatory bodies reviewing the research. While informed consent begins to address the interests of research participants through the disclosure of materially relevant information and due attention to adequate comprehension and voluntariness, it is not designed to address the full range of legitimate interests in the trial. It seldom extends beyond the highly structured and managed interactions of the consent processes, and does not attempt to elicit the prospective research participants’ perspectives about the research more broadly, i.e., beyond the immediate implications of their participation, to the implications for their lives and their communities. However, in drawing attention to these broader interests of the research community, CE may also offer insights that could be used to strengthen individual informed consent.

But the range of interests at stake in any research project go beyond those of the individual research participants, and therefore the relationships relevant to global health research extend beyond those between researchers and research participants as currently defined by regulations. If the ethical goals for CE, described above, reflect important ethical considerations that cannot be met through prospective review and individual informed consent alone, then researchers have a responsibility to establish relationships with the stakeholder community, which consists of those individuals or groups of individuals who have some interest or “stakes” in the research. Whereas the emphasis in research ethics has been squarely on risks and potential benefits associated with *participation* in research, with only limited attention to implications for “third parties”, this does not exhaust the full range of interests that are brought into play when a research project is undertaken in a host community.

Many groups and individuals will have interests at stake in the conduct and/or outcomes of a given research project, and not all can be actively engaged. A central task of CE is to identify the range of stakeholders for a given study, evaluate their potential interests, and prioritize whom to engage. Initially, that prioritization is informed by how and to what extent the proposed research will affect a stakeholder’s interests. The more significantly research affects the interests of a given stakeholder, the higher the priority of engaging that stakeholder. In addition, the responsibility to avoid harming others is generally stronger than the obligation to benefit them, so priority is given to engagement with stakeholders at risk of harm from the research. Inevitably, the manner and extent of engagement with each party will be subject to practical limits, including geographic dispersal and budgetary constraints [18].

This concept of the stakeholder community is broader than the traditional view of community in biomedical research, which has focused on pre-existing associations of individuals such as those living in the same geographic area, or sharing an ethnic, cultural, religious or occupational identity [26]. Pre-existing associations are only one way in which the interests of individuals collect. For any given research project, individual stakeholders may have dramatically different interests at stake in its conduct and/or outcomes, but the fact that each holds some interests defines the stakeholder community, and grounds the moral responsibility for engagement. CE creates the human infrastructure necessary to support the deliberations and discussions that are necessary to discover, and to be responsive to, this range of interests.

## Summary

CE offers a promising solution to many seemingly intractable challenges in global health research by creating the infrastructure that allows researcher to engage with the social and political context of biomedical research. Despite this promise, there are concerns about what makes engagement meaningful [10–14]. We have responded to those concerns by presenting an ethical framework for CE. We argue that the ethical value of CE lies in its contribution to: identifying, and facilitating responsiveness to, non-obvious risks; extending respect beyond individual research participants to the community of stakeholders, whose interests may be affected; and to building the legitimacy of the research project. We argue further that these ethical goals are realized primarily through a human infrastructure of relationships between researchers and stakeholders. There is on-going uncertainty about when CE should be recommended or required for the ethical conduct of research, [10, 11] an issue for which there remains frustratingly little guidance, in particular, for research ethics committees. CE activities, being resource-intensive, may not be necessary or feasible for many research projects, and issues of scale and efficiency are yet to be worked out. Nonetheless, the framework presented here advances that conversation by identifying the kinds of circumstances in which CE is important for the ethical conduct of research.

More specifically, this framework advances the theory and practice of community engagement in three key ways. First, it improves mindfulness among funders, investigators, research ethics committees, and relevant regulatory authorities about what CE practices aim to accomplish ethically, by improving the specificity and precision of the stated ethical goals. There is no shortage of literature addressing ethical aspects and implications of CE. CE has received extensive attention in many fields—e.g., anthropology, sociology, social psychology, environmental management, forestry, global health, global development, community-based participatory research, feminist philosophy, public engagement for new technology assessment, public health practice, and social network theory, among many others. Despite this rich and extensive literature, however, the specific ethical contribution of CE remains unclear [11]. By distilling these insights, the current framework aims to improve the ability of teams to plan, implement and review CE. Second, our framework helps research funders justify the allocation of funds to CE in research proposals by clarifying the potential value of the investment. Currently, there is little, if any, public discussion about how, and to what extent, CE should be funded, and no publicly available data—to the best of our knowledge—about costs associated with successful CE practices in research. Clarifying the ethical value proposition for CE might help move this issue forward on the research policy agenda. Third, our framework can make a modest contribution towards improving partnerships with host communities, by providing those communities with greater specificity and precision about what CE practices aim to achieve.

While this framework focuses on the goals of CE, it also sheds light on the question of when CE is important to the ethical conduct of research. In particular, this framework suggests that CE should be strongly considered when the conduct and outcomes of the proposed research affect the interests of groups and individuals who are not direct research participants. The greater the reach of the research beyond research participants, the greater the need for CE. Research projects vary in the extent to which they might affect the interests of parties beyond research participants. Certain kinds of trials, such as cluster-randomized trials, or trials of new broad-based public health interventions (e.g., insect vector control strategies), [27] reach far into the community, beyond individual research participants, and thereby warrant special attention in the form of CE. Of course, the need for CE is often identified in retrospect, after unforeseen harms have occurred, but having a framework that conceptualizes the contributions of CE more clearly may also help clarify the appropriate context of application.

Although we focus, in this paper, on global health research, we believe that these ethical goals of CE apply in any geographic setting. The ethical goals reflect quite fundamental human concerns and, therefore, we expect that they would not be easily subverted by cultural or political considerations. Indeed, in our own work, we have studied cases of CE in Canada and Australia in which these goals would clearly have been applicable.

CE represents an important means of improving both the ethics and practice of global biomedical research. Our effort to clarify the ethical goals of CE and to explore its contribution to the ethical conduct of research represent a first step in improving the recognition that biomedical research programs are ultimately human endeavors, and that a strong human infrastructure is critical to both their ethical and practical success.

## Abbreviations

CE: Community engagement
LMIC: Low and middle income countries.
